# Supplementary material for: Improved swin transformer-based thorax disease classification with optimal feature selection using chest X-ray
Source: PLoS One. 2025 Jun 25;20(6):e0327099. doi: 10.1371/journal.pone.0327099 (PMC12193078; doi:10.1371/journal.pone.0327099)
Supplement: Attachment — Submitted filename: Response to Reviewers Letter.docx [file pone.0327099.s001.docx]

**Response to Comments Made by the Editors and Reviewers**

**5. Review Comments to the Author**

Please use the space provided to explain your answers to the questions above. You may also include additional comments for the author, including concerns about dual publication, research ethics, or publication ethics. (Please upload your review as an attachment if it exceeds 20,000 characters)

**Reviewer 1’s comments to author:**

Review of the Paper: "Improved Swin Transformer based Thorax Disease Classification with Optimal Feature Selection using Chest X-ray"

The paper proposes a novel method for classifying thoracic diseases using a deep learning framework that combines an Improved Swin Transformer for classification, Chaotic Whale Optimization for feature selection, and Enhanced Auto-Encoder for feature extraction. However, there are some restrictions need to be solved.

1. There is uncertainty over the model's applicability in real-world clinical operations. There hasn't been any clinical validation with radiologists.

**Response:** We appreciate the reviewer’s observation regarding the lack of clinical validation. We acknowledge this as a critical step towards real-world applicability. Accordingly, we have explicitly addressed this limitation in the revised manuscript under the Future Work section, where we discuss the need for prospective validation in clinical settings, including collaboration with radiologists to assess the model’s performance and integration feasibility in actual workflows. The changes are highlighted red colour in the revised research manuscript in section 5.1. The changes are described as follows;

Thus, Future research should carry out comprehensive clinical validation tests with radiologists in order to overcome the uncertainties around the model's applicability in actual clinical situations. In order to assess the model's interpretability, dependability, and practical utility in routine diagnostics, such investigations are crucial. Refinements can be guided by collaborations with doctors to better address clinical demands. Trust and usability will be improved by incorporating explainability elements. Furthermore, deployment requires optimizing computing efficiency, particularly in environments with restricted resources. It will be easier to move from a research prototype to a clinically useful tool for diagnosing thorax disease if the dataset is expanded to include multi-institutional and multi-modal imaging data.

2. The proposed method only compares with limited number of state-of-the-art methods and also ignores newer methods.

**Response:** Thank you for this valuable comment. In response, we have updated the manuscript by including several recent and state-of-the-art methods in comparative study. This enhancement not only strengthens the experimental analysis but also better highlights the effectiveness of proposed approach. The changes are highlighted red colour in the revised research manuscript in section 4.7. The changes are described as follows;

**Table 6.** Comparative Analysis of proposed and state-of-the-art methods

| **Dataset 1** | | | |
| --- | --- | --- | --- |
| Methods/ Metrics | Accuracy | Precision | Recall |
| CX-Ultranet [30] | 0.844 | 0.857 | 0.835 |
| DenseNet121 [31] | 0.861 | 0.874 | 0.849 |
| CycleGAN [32] | 0.933 | 0.947 | 0.921 |
| **Proposed (90%)** | **0.964** | **0.977** | **0.9845** |
| **Dataset 2** | | | |
| CX-Ultranet | 0.842 | 0.832 | 0.819 |
| DenseNet121 | 0.867 | 0.869 | 0.842 |
| CycleGAN | 0.949 | 0.956 | 0.942 |
| **Proposed (90%)** | **0.986** | **0.985** | **0.985** |

A comparative analysis of proposed and state-of-the-art methods for classifying thoracic diseases across two datasets is shown in Table 6. The suggested approach achieves the highest accuracy, precision, and recall at 90%, outperforming all others. It considerably outperforms CycleGAN with 93.3% accuracy, 94.7% precision, and 92.1% recall on Dataset 1, achieving 96.4% accuracy, 97.7% precision, and 98.45% recall. Likewise, the suggested approach attains 98.6% accuracy, 98.5% precision, and 98.5% recall in Dataset 2. On the other hand, DenseNet121 and CX-Ultranet perform moderately, demonstrating the superior efficacy of the proposed model in classifying thoracic diseases.

3. There are different optimization algorithms that can be use instead of Chaotic Whale Optimization Algorithm. In the paper, do not mention any reason for using this algorithm. Additionally, ChWO algorithm adds extra computational overhead.

**Response:** We appreciate the reviewer’s valuable feedback. In response, we have updated the manuscript by providing a more detailed justification for the selection of the Chaotic Whale Optimization Algorithm (ChWO). Furthermore, we acknowledge the additional computational overhead introduced by ChWO. However, we believe this trade-off is justified by the observed improvement in solution quality and convergence speed, as demonstrated in our experimental results. The changes are highlighted red colour in the revised research manuscript in section 3.5. The changes are described as follows;

ChWO improves the randomization criteria by introducing chaotic Chebyshev mapping into the traditional whale optimization process. This lessens the possibility of being stuck in local optima, a typical drawback of many optimization strategies, and enables the algorithm to more efficiently traverse a larger search space. The whale optimization process's rate of convergence is accelerated by the chebyshev mapping. The ChWO ensures effective optimization during feature selection since it converges more quickly than the conventional whale optimization algorithm. By selecting the most relevant and ideal features from the extracted set, ChWO feature selection greatly enhances the classification of thoracic diseases.

4. There are different number of chest x-ray datasets that can be used for evaluating the approach. Using just one dataset is not enough.

**Response:** We appreciate the reviewer’s insightful comment regarding the use of multiple datasets for evaluation. In response, we have incorporated an additional dataset to strengthen the robustness and generalizability of our approach. The description of the newly added dataset has been included in Section 3.1, and the corresponding analysis and experimental results have been updated and discussed in Section 4. This addition provides a more comprehensive evaluation and further validates the effectiveness of our method across different data distributions. The changes are highlighted red colour in the revised research manuscript. The changes are described as follows;

**3.1 Data Acquisition**

The input is obtained from the publicly available dataset and is utilized for the evaluation of the proposed EnAE+ChWO+IMSTrans model. The datasets like Chest X-Ray dataset [27] and Lung Disease Dataset [35] are utilized for the evaluation of the proposed EnAE+ChWO+IMSTrans-based Thorax disease classification.

***ChestX-Ray dataset [D1]:*** Dataset 1 contains a total of 8,274 chest X-ray images in 1024×1024 PNG format, covering 14 disease classes: Atelectasis, Cardiomegaly, Effusion, Infiltration, Mass, Nodule, Pneumonia, Pneumothorax, Consolidation, Edema, Emphysema, Fibrosis, Pleural Thickening, and Hernia. Each image is accompanied by rich metadata (from Data_Entry_2017.csv) including patient age, gender, view position, and more. This dataset provides a realistic and challenging basis for evaluating computer-aided diagnosis systems due to the visual similarity of pathologies and the complexity of clinical interpretation, even in comparison to CT imaging.

***Lung Disease Dataset [D2]:*** This dataset has been created using the NIH CXR Structured Dataset 248, which contains Chest X-ray (CXR) images aimed at supporting the classification of lung diseases. This dataset contains a total of 4,260 high-resolution chest X-ray images, categorized into three classes: Normal Lungs, Fibrosis Lungs, and Pneumonia Lungs. The images are organized into separate folders for each category to ensure structured access and labeling.

**4.1 Dataset Description**

The datasets like ChestX-Ray dataset [27] and Lung Disease Dataset [35] are utilized for the evaluation of the proposed EnAE+ChWO+IMSTrans-based Thorax disease classification.

**4.1.1 Original Data Distribution:**

**Dataset 1: ChestX-Ray dataset**

- The dataset consists of 8,274 chest X-ray images across 14 thoracic disease categories:
- Atelectasis, Cardiomegaly, Effusion, Infiltration, Mass, Nodule, Pneumonia, Pneumothorax, Consolidation, Edema, Emphysema, Fibrosis, Pleural Thickening, and Hernia.
- These images are annotated with one or more disease labels, and the dataset is inherently multi-label and imbalanced. For instance, diseases such as Infiltration and Effusion are more frequent, while Hernia and Fibrosis are rare.
- This imbalance was initially visualized through class-wise frequency plots to better understand the skewness in data distribution.

**Dataset 2: Lung Disease Dataset**

- This dataset contains 4,260 high-resolution chest X-ray images organized into three classes:
- Normal Lungs (no abnormalities),
- Fibrosis Lungs and
- Pneumonia Lungs.
- The data also exhibits imbalance, with Normal cases being more prevalent compared to Fibrosis and Pneumonia.

**4.1.2 Data Distributions and Preprocessing Steps**

**Dataset 1: ChestX-Ray dataset**

- **Source**: Publicly available NIH ChestX-ray14 dataset.
- **Subset Size Used**: 8,274 chest X-ray images.
- **Classes** (14 in total):
  Atelectasis, Cardiomegaly, Effusion, Infiltration, Mass, Nodule, Pneumonia, Pneumothorax, Consolidation, Edema, Emphysema, Fibrosis, Pleural Thickening, Hernia.
- **Nature**: Multi-label, class-imbalanced dataset.
  Infiltration and Effusion are overrepresented, while Hernia and Fibrosis are rare.
- **Label Handling**: The "Finding Labels" column is parsed to assign binary multi-label vectors for each class.
- **Preprocessing:**
  - Images with missing or unreadable files were removed.
  - Images resized to 256×256, followed by random crop (224×224).
  - Pixel normalization with ImageNet statistics.
  - Missing or null labels were excluded.
- **Data Augmentation**:
  - Random horizontal and vertical flips (p=0.5).
  - Random rotations (up to ±30 degrees).
  - Augmentation increases feature variability but does not alter label distribution.

**Dataset 2: Lung Disease Dataset**

- **Source**: dataset containing **4,260 images**.
- **Classes**: Normal, Pneumonia, and Fibrosis.
- **Nature**: Multi-label, class-imbalanced dataset.
- **Preprocessing:**
  - Images resized and normalized using the same procedure as Dataset 1 to ensure consistency.
  - Verified for label completeness and removed any ambiguous or mislabeled samples.

**4.1.3 Data Augmentation and Impact:**

To address the data imbalance and enhance model generalization, applied a robust augmentation pipeline during training that includes:

- Spatial transformations (resizing, random cropping),
- Geometric variations (horizontal/vertical flipping, random rotations),
- Normalization to standardize image intensity.

These augmentations are applied dynamically at each training epoch. While the underlying image count remains unchanged, the model is exposed to diverse representations of each sample, especially from minority classes. This synthetically balances the training data, increases intra-class variance, and reduces overfitting.

**4.1.4 Hyperparameter Tuning and Cross-Validation:**

Enhancing the input feature space through feature selection based on the ChWO algorithm was the optimization strategy's main objective. By making sure that only the most relevant features were included for classification, this technique successfully decreased feature redundancy, increased class separability, and improved overall model performance.

- **Batch Size and Learning Rate:** To balance memory limitations with training stability, we chose widely used empirically validated settings a fixed learning rate of 1e-4 and a batch size of 32.
- **Optimizer:** The Adam optimizer was chosen because of its demonstrated convergence efficiency in deep learning problems, and it has a weight decay of 1e-5.
- **Training Epochs:** Up to 100 training epochs were used to give the models enough time to converge without being overfit.

**4.2 Comparative Assessment**

The comparative assessment is devised using two various datasets like D1 and D2 with the existing Thorax disease detection methods like ThoraX-PriorNet [21], CXray-EffDet [22], Attention-based CNN [23], and Swin Transformer [26]. The definition for the assessment measures is portrayed in Table 2.

**Table 2:** Definition for Assessment Measures

| **Measures** | **Formula** | **Symbol Definition** |
| --- | --- | --- |
| **Accuracy** | $\frac{TP+TN}{TP+TN+FP+FN}$ | $TP$True Positive  $TN$True Negative  $FP$False Positive  $FN$False Negative |
| **Precision** | $\frac{TP}{TP+FP}$ |  |
| **Recall** |  |  |
| **F-Measure** | $2\times\frac{\Pr ecision\times\mathrm{Re}call}{\Pr ecision+\mathrm{Re}call}$ |  |
| **MCC** | $MCC=\frac{TP\cdot TN-FP\cdot FN}{\sqrt{\left(TP+FP\right)\left(TP+FN\right)\left(TN+FP\right)\left(TN+FN\right)}}$ |  |
| **MAE** |  | $\hat{y}_{i}$-predicted value  $y_{i}$-actual value  $n$-number of observation |

**4.2.1 Assessment Using Dataset 1**

The assessment of Thorax disease classification method using the D1 dataset is evaluated based on training data and is elaborated in this section.

The accuracy-based performance evaluation of several models at varying training percentages is displayed in Figure 6. Accuracy defines the proportion of correct predictions made by the model out of the total samples. The accuracy assessment of Thorax disease classification methods is portrayed in Figure 6 (a). Here, the EnAE is key to extracting critical attributes using stacked auto-encoders combined with an attention module, EnAE extracts important features, removing noise and irrelevant data. In addition, IMSTrans leverages advanced attention mechanisms and hierarchical feature extraction, allowing it to process image patches efficiently. With values ranging from 0.878 at 50% training to 0.964 at 90%, the suggested model achieves the highest accuracy and routinely outperforms alternative approaches. Thus, the proposed EnAE+ChWO+IMSTrans model captures both local and global patterns in the X-rays, which leads to more accurate predictions compared to existing approaches. The precision analysis portrayed in Figure 6 (b). Here, the ChWO algorithm utilized to select the most significant features after extraction. By discarding less relevant attributes, the model is streamlined to focus on features that are strongly correlated with specific thoracic diseases, reducing the risk of making incorrect classifications. The model's highly accurate performance in predicting positives is demonstrated by the proposed method's highest precision of 0.977 at 90% training percentage. Thus, the incorrect classifications are eliminated by the proposed model that leads to higher precision by the proposed EnAE+ChWO+IMSTrans model. The recall-based analysis is demonstrated in Figure 6 (c) The proposed method achieves higher recall due to the balanced data and efficient feature extraction modules. By augmenting the minority class samples, the model becomes better at identifying true cases of Thorax diseases that assist in improving its recall. In addition, the EnAE captures finer and more complex features, ensuring that the model can detect even minor indicators of thoracic diseases, reducing false negatives. With the highest recall of 0.9845 at 90% training percentage, the suggested technique accurately identify the majority of relevant instances.

|  |  |
| --- | --- |
| **(a)** | **(b)** |
|  |  |
| **(c)** | **(d)** |
|  |  |
| **(e)** | **(f)** |

**Figure 6:** Assessment Using Dataset 1: (a) Accuracy, (b) Precision, (c) Recall, (d) F-Measure, (e) MCC and (f) MAE

The F1-score-based analysis is demonstrated in Figure 6 (d) The EnAE’s ability to extract significant features and IMSTrans’s powerful classification ensure that both precision and recall are maximized. It indicates that the proposed model helps accomplish both sensitive in detecting diseases (high recall) and specificity in making correct predictions (high precision). The maximum F1-Score of 0.964 is obtained by the proposed method at 90% training percentage, demonstrating an effective trade-off between recall and precision. Matthews Correlation Coefficient (MCC) is measured to analyze the robustness of the Thorax disease for both positive and negative classes. The MCC-based analysis is demonstrated in Figure 6 (e). The use of data augmentation helps reduce the bias toward majority classes that results in a more balanced classification. It leads to lower false positive and false negative rates, which contribute to a higher MCC score by the proposed EnAE+ChWO+IMSTrans model. The proposed method achieves the maximum MCC of 0.9647 at 90%, indicating that it is the most dependable and balanced model. The MAE-based analysis is demonstrated in Figure 6 (f). EnAE minimizes reconstruction error when extracting features, which ensures that the latent representations of the X-rays are highly accurate. It reduces the overall error in classifying the Thorax disease. The careful selection of features helps minimize prediction errors. By ensuring that only relevant features are used, the model avoids making large errors, which keeps the MAE low. The proposed method has the lowest MAE of 0.184 at 90%, meaning it predicts with the least amount of error. Table 3 shows the Assessment Using Dataset 1

**Table 3.** Assessment Using Dataset 1

| Methods/ Training  Percentage | 50 | 60 | 70 | 80 | 90 |
| --- | --- | --- | --- | --- | --- |
| Accuracy | | | | | |
| ThoraX-PriorNet | 0.806 | 0.812 | 0.843 | 0.884 | 0.887 |
| CXray-EffDet | 0.813 | 0.822 | 0.835 | 0.905 | 0.912 |
| Attention based CNN | 0.836 | 0.834 | 0.846 | 0.891 | 0.928 |
| Swin Transformer | 0.842 | 0.844 | 0.851 | 0.913 | 0.945 |
| **Proposed** | **0.878** | **0.896** | **0.909** | **0.92** | **0.964** |
| **Precision** | | | | | |
| ThoraX-PriorNet | 0.8265 | 0.8705 | 0.876 | 0.8942 | 0.9115 |
| CXray-EffDet | 0.8452 | 0.872 | 0.8885 | 0.94 | 0.944 |
| Attention based CNN | 0.891 | 0.917 | 0.9255 | 0.929 | 0.958 |
| Swin Transformer | 0.8737 | 0.8794 | 0.9078 | 0.9239 | 0.9534 |
| **Proposed** | **0.896** | **0.903** | **0.931** | **0.9515** | **0.977** |
| **Recall** | | | | | |
| ThoraX-PriorNet | 0.778 | 0.7455 | 0.806 | 0.8825 | 0.8715 |
| CXray-EffDet | 0.7925 | 0.77 | 0.7865 | 0.867 | 0.874 |
| Attention based CNN | 0.7855 | 0.772 | 0.785 | 0.8915 | 0.931 |
| Swin Transformer | 0.8256 | 0.8597 | 0.8662 | 0.8926 | 0.9578 |
| **Proposed** | **0.855** | **0.8815** | **0.8935** | **0.921** | **0.9845** |
| **F1-Score** | | | | | |
| ThoraX-PriorNet | 0.8023 | 0.8041 | 0.8392 | 0.8885 | 0.892 |
| CXray-EffDet | 0.8178 | 0.8195 | 0.838 | 0.9045 | 0.9118 |
| Attention based CNN | 0.8367 | 0.8385 | 0.8502 | 0.8955 | 0.9337 |
| Swin Transformer | 0.8481 | 0.8712 | 0.8842 | 0.9089 | 0.9423 |
| **Proposed** | **0.8768** | **0.894** | **0.9125** | **0.9385** | **0.964** |
| **MCC** | | | | | |
| ThoraX-PriorNet | 0.7793 | 0.7476 | 0.8102 | 0.8827 | 0.8743 |
| CXray-EffDet | 0.7924 | 0.7712 | 0.7888 | 0.8697 | 0.8771 |
| Attention based CNN | 0.8074 | 0.8128 | 0.8887 | 0.8914 | 0.9428 |
| Swin Transformer | 0.8355 | 0.8605 | 0.8686 | 0.8976 | 0.9368 |
| **Proposed** | **0.8558** | **0.8819** | **0.8971** | **0.9242** | **0.9647** |
| **MAE** | | | | | |
| ThoraX-PriorNet | 0.2585 | 0.2354 | 0.2218 | 0.2182 | 0.2148 |
| CXray-EffDet | 0.2473 | 0.2326 | 0.2237 | 0.2161 | 0.2106 |
| Attention based CNN | 0.2434 | 0.231 | 0.2169 | 0.2164 | 0.2049 |
| Swin Transformer | 0.2401 | 0.2226 | 0.2137 | 0.2129 | 0.2001 |
| **Proposed** | **0.233** | **0.2143** | **0.2048** | **0.2017** | **0.184** |

**4.2.1 Assessment Using Dataset 2**

The assessment of Thorax disease classification method using the D2 dataset is evaluated based on training data and is elaborated in this section.

|  |  |
| --- | --- |
| **(a)** | **(b)** |
|  |  |
| **(c)** | **(d)** |
|  |  |
| **(e)** | **(f)** |

**Figure 7:** Assessment Using Dataset 2: (a) Accuracy, (b) Precision, (c) Recall, (d) F-Measure, (e) MCC and (f) MAE

Figure 7 analyzes the performance of various Thorax disease classification techniques on the D2 dataset, comparing their efficacy at different training data proportions, ranging from 50% to 90%. The accuracy is shown in 7(a), where EnaE+CHWO+IMSTrans continuously attains the best accuracy, hitting roughly 0.96 at 90% training data. Likewise, in 7 (b) Precision, EnaE+CHWO+IMSTrans is in the lead with approximately 0.95 at 90%. In 7(c), the Recall exhibits a comparable pattern, with EnaE+CHWO+IMSTrans reaching roughly 0.94. The F-Measure in 7(d), which strikes a balance between recall and precision, likewise peaks for EnaE+CHWO+IMSTrans at about 0.94. Figure 7 (e) shows that the MCC scores for all models generally improve as the training percentage rises from 50% to 90%. In particular, proposed EnAe+CHWO+IMSTrans attains the greatest MCC with 90% training data, at roughly 0.96.  At the maximum training , ThoraX-PriorNet has the lowest MCC, circling 0.86. This suggests that, particularly when trained on more data, EnAe+CHWO+IMSTrans have the greatest overall classification performance. Figure (f) shows that the MAE for all models tends to drop as the training rises. EnAe+CHWO+IMSTrans shows the lowest MAE at the 90% training level, which is at 0.04, indicating the smallest average magnitude of errors in its predictions. At 90% training data, ThoraX-PriorNet has the greatest MAE, approximately 0.09, indicating higher average prediction errors. This suggests that, when trained with a larger dataset, EnAe+CHWO+IMSTrans produces the most accurate numerical predictions out of all the models that were compared. Table 4 shows the assessment using Dataset 2.

**Table 4.** Assessment Using Dataset 2

| Methods/ Training  Percentage | 50 | 60 | 70 | 80 | 90 |
| --- | --- | --- | --- | --- | --- |
| Accuracy | | | | | |
| ThoraX-PriorNet | 0.823 | 0.828 | 0.853 | 0.903 | 0.91 |
| CXray-EffDet | 0.804 | 0.841 | 0.868 | 0.901 | 0.927 |
| Attention based CNN | 0.845 | 0.85 | 0.902 | 0.913 | 0.946 |
| Swin Transformer | 0.862 | 0.861 | 0.887 | 0.926 | 0.959 |
| **Proposed** | **0.893** | **0.908** | **0.926** | **0.944** | **0.986** |
| **Precision** | | | | | |
| ThoraX-PriorNet | 0.817 | 0.859 | 0.872 | 0.892 | 0.918 |
| CXray-EffDet | 0.841 | 0.885 | 0.894 | 0.909 | 0.936 |
| Attention based CNN | 0.858 | 0.891 | 0.913 | 0.942 | 0.955 |
| Swin Transformer | 0.876 | 0.885 | 0.931 | 0.926 | 0.962 |
| **Proposed** | **0.899** | **0.911** | **0.941** | **0.963** | **0.985** |
| **Recall** | | | | | |
| ThoraX-PriorNet | 0.784 | 0.742 | 0.789 | 0.862 | 0.877 |
| CXray-EffDet | 0.797 | 0.788 | 0.804 | 0.856 | 0.879 |
| Attention based CNN | 0.823 | 0.838 | 0.841 | 0.903 | 0.9 |
| Swin Transformer | 0.876 | 0.899 | 0.91 | 0.897 | 0.926 |
| **Proposed** | **0.883** | **0.902** | **0.932** | **0.946** | **0.985** |
| **F1-Score** | | | | | |
| ThoraX-PriorNet | 0.801 | 0.81 | 0.843 | 0.876 | 0.89 |
| CXray-EffDet | 0.822 | 0.834 | 0.834 | 0.887 | 0.913 |
| Attention based CNN | 0.846 | 0.849 | 0.867 | 0.9 | 0.942 |
| Swin Transformer | 0.833 | 0.851 | 0.873 | 0.92 | 0.94 |
| **Proposed** | **0.872** | **0.897** | **0.917** | **0.946** | **0.993** |
| **MCC** | | | | | |
| ThoraX-PriorNet | 0.779 | 0.762 | 0.825 | 0.874 | 0.877 |
| CXray-EffDet | 0.801 | 0.797 | 0.814 | 0.859 | 0.9 |
| Attention based CNN | 0.829 | 0.831 | 0.894 | 0.903 | 0.942 |
| Swin Transformer | 0.823 | 0.848 | 0.883 | 0.911 | 0.942 |
| **Proposed** | **0.861** | **0.887** | **0.903** | **0.935** | **0.978** |
| **MAE** | | | | | |
| ThoraX-PriorNet | 0.177 | 0.172 | 0.147 | 0.097 | 0.09 |
| CXray-EffDet | 0.196 | 0.159 | 0.132 | 0.099 | 0.073 |
| Attention based CNN | 0.155 | 0.15 | 0.098 | 0.087 | 0.054 |
| Swin Transformer | 0.138 | 0.139 | 0.113 | 0.074 | 0.041 |
| **Proposed** | **0.107** | **0.092** | **0.074** | **0.056** | **0.014** |

**4.3 Accuracy-Loss:** Accuracy-loss analysis compares the model's accuracy and loss during both training and testing phases to assess the performance of the model and determine if it's generalizing well to unseen data.

The accuracy and loss trends for Dataset 1 to classify thorax disease over 100 epochs are displayed in Figure 8. Over 100 epochs, the accuracy trends are shown in Figure 8(a). The accuracy of training rises quickly, peaking at about 95% around epoch 30 before plateauing. The trajectory of the testing accuracy is similar, with modest swings after a slightly lower peak at 94%. This shows that the model is capable of learning and generalizing to new data with reasonable accuracy. In Figure 8(b), the loss curves are shown. Over time, the training loss decreases from roughly 0.7 to less than 0.2. After a brief period of volatility, the testing loss likewise drops dramatically, stabilizing at about 0.2. It is clear from the rather narrow difference between training and testing loss that the model is not significantly overfitting the training set.

|  |  |
| --- | --- |
| (a) | (b) |

**Figure 8.** Accuracy-Loss Analysis for Dataset 1

**Figure 9.** Accuracy-Loss Analysis for Dataset 2

The accuracy and loss trends for Dataset 2 to classify thorax disease over 100 epochs are displayed in Figure 9. Minor overfitting is suggested by the testing loss, which drops to a minimum of 0.21 before slightly increasing to 0.22, while the training loss gradually reduces from roughly 0.7 to 0.19. On the other hand, the black training accuracy quickly increases from 0.5 to a plateau at 0.97. Also improving is the testing accuracy, which peaks at about 0.95 and stays steady. Although there is some overfitting in later epochs, the model's overall high testing accuracy of almost 95% shows that learning is effective.

**4.4 Confusion Matrix:** The confusion matrix representation of the proposed EnAE+ChWO+IMSTrans model using dataset 1 and dataset 2 is portrayed in Figure 10, which illustrates the correct classification made by the proposed method.

|  |  |
| --- | --- |
| (a) | (b) |

**Figure 10**: Confusion Matrix for (a) Dataset 1 and (b) Dataset 2

Figure 10 (a) shows a 14x14 matrix for thorax disease classification. For a number of classes, including class 0 (82), class 2 (71), and class 4 (107), the diagonal displays high correct classifications. Misclassifications sometimes occur, though; for example, 37 cases of class 1 were incorrectly classified as class 0. The three categories of fibrosis, normal, and pneumonia are simplified in Figure 10 (b). With 250 pneumonia cases correctly predicted, 201 normal cases correctly classified, and 573 fibrosis patients correctly recognized, the matrix shows strong results. Three cases of pneumonia were misclassified as fibrosis, while two cases of normal were misclassified as fibrosis. At 0.986, this classification's overall accuracy score on Dataset 2 is remarkably high. The model's accuracy in classifying thoracic disorders is clearly demonstrated by these matrices, with Dataset 2 exhibiting especially strong findings.

**4.5 AUC Analysis:** The AUC analysis of Dataset 1 is portrayed in Figure 11. The area under the ROC curve is ranging from 0 to 1. A higher AUC value by EnAE+ChWO+IMSTrans model indicates better performance in distinguishing between classes. The analysis of Dataset 2's Area Under the Curve (AUC) is shown in Figure 12. Various model's performances are evaluated in 12 (a), and the model with the greatest AUC, EnAE+ClWO+IMSTrans, shows superior classification abilities. The AUC curves for several classes (0, 1, and 2) are shown in 12(b). All of these classes attain unusually high AUC scores of 0.99, indicating superior discrimination ability across all Dataset 2 categories.

|  |  |
| --- | --- |
| (a) | (b) |

**Figure 11.** AUC Analysis for Dataset 1

|  |  |
| --- | --- |
| (a) | (b) |

**Figure 12.** AUC Analysis for Dataset 2

**4.6 Convergence Analysis:** Convergence analysis evaluates how quickly and effectively an optimization algorithm minimizes the loss function, ensuring that the model reaches the global or near-global minimum efficiently. The convergence analysis is demonstrated in Figure 13. The inclusion of chaotic mapping assist the proposed ChWO algorithm to converge faster compared to existing whale optimization algorithm.

**Figure 13.** Convergence Analysis

**4.7 Comparative Discussion**

The comparative discussion for Dataset 1 and Dataset 2 based on the best case with 90% of training data and 10% of testing data is portrayed in Table 5. The proposed EnAE+ChWO+IMSTrans approach performs better across all assessment measures, according to a comparative analysis of Thorax disease categorization on Datasets 1 and 2. The proposed approach outperformed ThoraX-PriorNet, CXray-EffDet, Attention-based CNN, and Swin Transformer by 7.99%, 5.39%, 3.73%, and 1.97%, respectively, and obtained the highest accuracy (0.964) for Dataset 1. Likewise, the proposed technique outperformed Swin Transformer, the next best method, by 2.81% for Dataset 2, achieving an accuracy of 0.986. Throughout both datasets, the proposed approach continuously produced superior outcomes in terms of accuracy, recall, F-score, and MCC. Its robustness in detecting true positives is noteworthy, as evidenced by the recall on Datasets 1 (0.9845) and 2 (0.985). Additionally, the model's Mean Absolute Error (MAE) was the lowest, particularly on Dataset 2, at 0.014, which is far lower than all baseline techniques. These findings highlight the improved generality and efficacy of the proposed method in classifying Thorax disease, particularly when 90% of the data is used for training and 10% is used for testing. Overall, the two dataset's consistent superiority highlights its appropriateness for dependable clinical use in medical image classification tasks.

**Table 5.** Comparative Analysis for Dataset 1 and Dataset 2

| Methods/ Metrics | Accuracy | Precision | Recall | F-Score | MCC | MAE |
| --- | --- | --- | --- | --- | --- | --- |
| Dataset 1 | | | | | | |
| ThoraX-PriorNet | 0.887 | 0.9115 | 0.8715 | 0.892 | 0.8743 | 0.2148 |
| CXray-EffDet | 0.912 | 0.944 | 0.874 | 0.9118 | 0.8771 | 0.2106 |
| Attention based CNN | 0.928 | 0.958 | 0.931 | 0.9337 | 0.9428 | 0.2049 |
| Swin Transformer | 0.945 | 0.9534 | 0.9578 | 0.9423 | 0.9368 | 0.2001 |
| **Proposed** | **0.964** | **0.977** | **0.9845** | **0.964** | **0.9647** | **0.184** |
| Dataset 2 | | | | | | |
| ThoraX-PriorNet | 0.91 | 0.918 | 0.877 | 0.89 | 0.877 | 0.09 |
| CXray-EffDet | 0.927 | 0.936 | 0.879 | 0.913 | 0.9 | 0.073 |
| Attention based CNN | 0.946 | 0.955 | 0.9 | 0.942 | 0.942 | 0.054 |
| Swin Transformer | 0.959 | 0.962 | 0.926 | 0.94 | 0.942 | 0.041 |
| **Proposed** | **0.986** | **0.985** | **0.985** | **0.993** | **0.978** | **0.014** |

Here, the comparative analysis portrays the superiority of the proposed model compared to the existing methods.

**Table 6.** Comparative Analysis of proposed and state-of-the-art methods

| **Dataset 1** | | | |
| --- | --- | --- | --- |
| Methods/ Metrics | Accuracy | Precision | Recall |
| CX-Ultranet [30] | 0.844 | 0.857 | 0.835 |
| DenseNet121 [31] | 0.861 | 0.874 | 0.849 |
| CycleGAN [32] | 0.933 | 0.947 | 0.921 |
| **Proposed (90%)** | **0.964** | **0.977** | **0.9845** |
| **Dataset 2** | | | |
| CX-Ultranet | 0.842 | 0.832 | 0.819 |
| DenseNet121 | 0.867 | 0.869 | 0.842 |
| CycleGAN | 0.949 | 0.956 | 0.942 |
| **Proposed (90%)** | **0.986** | **0.985** | **0.985** |

A comparative analysis of proposed and state-of-the-art methods for classifying thoracic diseases across two datasets is shown in Table 6. The suggested approach achieves the highest accuracy, precision, and recall at 90%, outperforming all others. It considerably outperforms CycleGAN with 93.3% accuracy, 94.7% precision, and 92.1% recall on Dataset 1, achieving 96.4% accuracy, 97.7% precision, and 98.45% recall. Likewise, the suggested approach attains 98.6% accuracy, 98.5% precision, and 98.5% recall in Dataset 2. On the other hand, DenseNet121 and CX-Ultranet perform moderately, demonstrating the superior efficacy of the proposed model in classifying thoracic diseases.

5. The dataset size and detailed information about it are not fully discussed.

**Response:** Thank you for pointing this out. We have now added a comprehensive description of the dataset in the revised manuscript. This includes the total number of samples, the distribution across classes, data collection methodology, preprocessing steps, and relevant statistical summaries. The changes are highlighted red colour in the revised research manuscript in section 4.1. The changes are described as follows;

**4.1 Dataset Description**

The datasets like ChestX-Ray dataset [27] and Lung Disease Dataset [35] are utilized for the evaluation of the proposed EnAE+ChWO+IMSTrans-based Thorax disease classification.

**4.1.1 Original Data Distribution:**

**Dataset 1: ChestX-Ray dataset**

- The dataset consists of 8,274 chest X-ray images across 14 thoracic disease categories:
- Atelectasis, Cardiomegaly, Effusion, Infiltration, Mass, Nodule, Pneumonia, Pneumothorax, Consolidation, Edema, Emphysema, Fibrosis, Pleural Thickening, and Hernia.
- These images are annotated with one or more disease labels, and the dataset is inherently multi-label and imbalanced. For instance, diseases such as Infiltration and Effusion are more frequent, while Hernia and Fibrosis are rare.
- This imbalance was initially visualized through class-wise frequency plots to better understand the skewness in data distribution.

**Dataset 2: Lung Disease Dataset**

- This dataset contains 4,260 high-resolution chest X-ray images organized into three classes:
- Normal Lungs (no abnormalities),
- Fibrosis Lungs and
- Pneumonia Lungs.
- The data also exhibits imbalance, with Normal cases being more prevalent compared to Fibrosis and Pneumonia.

**4.1.2 Data Distributions and Preprocessing Steps**

**Dataset 1: ChestX-Ray dataset**

- **Source**: Publicly available NIH ChestX-ray14 dataset.
- **Subset Size Used**: 8,274 chest X-ray images.
- **Classes** (14 in total):
  Atelectasis, Cardiomegaly, Effusion, Infiltration, Mass, Nodule, Pneumonia, Pneumothorax, Consolidation, Edema, Emphysema, Fibrosis, Pleural Thickening, Hernia.
- **Nature**: Multi-label, class-imbalanced dataset.
  Infiltration and Effusion are overrepresented, while Hernia and Fibrosis are rare.
- **Label Handling**: The "Finding Labels" column is parsed to assign binary multi-label vectors for each class.
- **Preprocessing:**
  - Images with missing or unreadable files were removed.
  - Images resized to 256×256, followed by random crop (224×224).
  - Pixel normalization with ImageNet statistics.
  - Missing or null labels were excluded.
- **Data Augmentation**:
  - Random horizontal and vertical flips (p=0.5).
  - Random rotations (up to ±30 degrees).
  - Augmentation increases feature variability but does not alter label distribution.

**Dataset 2: Lung Disease Dataset**

- **Source**: dataset containing **4,260 images**.
- **Classes**: Normal, Pneumonia, and Fibrosis.
- **Nature**: Multi-label, class-imbalanced dataset.
- **Preprocessing:**
  - Images resized and normalized using the same procedure as Dataset 1 to ensure consistency.
  - Verified for label completeness and removed any ambiguous or mislabeled samples.

**4.1.3 Data Augmentation and Impact:**

To address the data imbalance and enhance model generalization, applied a robust augmentation pipeline during training that includes:

- Spatial transformations (resizing, random cropping),
- Geometric variations (horizontal/vertical flipping, random rotations),
- Normalization to standardize image intensity.

These augmentations are applied dynamically at each training epoch. While the underlying image count remains unchanged, the model is exposed to diverse representations of each sample, especially from minority classes. This synthetically balances the training data, increases intra-class variance, and reduces overfitting.

6. The best results in the tables do not bold.

**Response:** We appreciate the reviewer’s comment. We have carefully reviewed the tables and have now bolded the best results for clarity and consistency. The changes are highlighted red colour in the revised research manuscript. The changes are described as follows;

7. Overall, the article is not well-published and well-structured, especially in the Result and Discussion section.

**Response:** We appreciate the reviewer’s feedback regarding the structure of the article, particularly the Results and Discussion section. In response, we have thoroughly revised this section and enhanced clarity and coherence. Specifically, we have included a detailed performance analysis to strengthen the interpretation of results. Additionally, the figures and tables have been updated, and their numbering has been rearranged accordingly to improve the overall flow and readability of the manuscript. The changes are highlighted red colour in the revised research manuscript in section 4. The changes are described as follows;

**4. Result and Discussion**

The implementation of the proposed EnAE+ChWO+IMSTrans-based Thorax disease classification is employed in PYTHON programming language. The analysis is devised for various training and testing percentages and the outcome derived by the testing is presented in this section. The proposed method is compared with existing Thorax disease detection methods like ThoraX-PriorNet [21], CXray-EffDet [22], Attention-based CNN [23], and Swin Transformer [26] to portray the superiority of EnAE+ChWO+IMSTrans based Thorax disease classification model.

**4.1 Dataset Description**

The datasets like ChestX-Ray dataset [27] and Lung Disease Dataset [35] are utilized for the evaluation of the proposed EnAE+ChWO+IMSTrans-based Thorax disease classification.

**4.1.1 Original Data Distribution:**

**Dataset 1: ChestX-Ray dataset**

- The dataset consists of 8,274 chest X-ray images across 14 thoracic disease categories:
- Atelectasis, Cardiomegaly, Effusion, Infiltration, Mass, Nodule, Pneumonia, Pneumothorax, Consolidation, Edema, Emphysema, Fibrosis, Pleural Thickening, and Hernia.
- These images are annotated with one or more disease labels, and the dataset is inherently multi-label and imbalanced. For instance, diseases such as Infiltration and Effusion are more frequent, while Hernia and Fibrosis are rare.
- This imbalance was initially visualized through class-wise frequency plots to better understand the skewness in data distribution.

**Dataset 2: Lung Disease Dataset**

- This dataset contains 4,260 high-resolution chest X-ray images organized into three classes:
- Normal Lungs (no abnormalities),
- Fibrosis Lungs and
- Pneumonia Lungs.
- The data also exhibits imbalance, with Normal cases being more prevalent compared to Fibrosis and Pneumonia.

**4.1.2 Data Distributions and Preprocessing Steps**

**Dataset 1: ChestX-Ray dataset**

- **Source**: Publicly available NIH ChestX-ray14 dataset.
- **Subset Size Used**: 8,274 chest X-ray images.
- **Classes** (14 in total):
  Atelectasis, Cardiomegaly, Effusion, Infiltration, Mass, Nodule, Pneumonia, Pneumothorax, Consolidation, Edema, Emphysema, Fibrosis, Pleural Thickening, Hernia.
- **Nature**: Multi-label, class-imbalanced dataset.
  Infiltration and Effusion are overrepresented, while Hernia and Fibrosis are rare.
- **Label Handling**: The "Finding Labels" column is parsed to assign binary multi-label vectors for each class.
- **Preprocessing:**
  - Images with missing or unreadable files were removed.
  - Images resized to 256×256, followed by random crop (224×224).
  - Pixel normalization with ImageNet statistics.
  - Missing or null labels were excluded.
- **Data Augmentation**:
  - Random horizontal and vertical flips (p=0.5).
  - Random rotations (up to ±30 degrees).
  - Augmentation increases feature variability but does not alter label distribution.

**Dataset 2: Lung Disease Dataset**

- **Source**: dataset containing **4,260 images**.
- **Classes**: Normal, Pneumonia, and Fibrosis.
- **Nature**: Multi-label, class-imbalanced dataset.
- **Preprocessing:**
  - Images resized and normalized using the same procedure as Dataset 1 to ensure consistency.
  - Verified for label completeness and removed any ambiguous or mislabeled samples.

**4.1.3 Data Augmentation and Impact:**

To address the data imbalance and enhance model generalization, applied a robust augmentation pipeline during training that includes:

- Spatial transformations (resizing, random cropping),
- Geometric variations (horizontal/vertical flipping, random rotations),
- Normalization to standardize image intensity.

These augmentations are applied dynamically at each training epoch. While the underlying image count remains unchanged, the model is exposed to diverse representations of each sample, especially from minority classes. This synthetically balances the training data, increases intra-class variance, and reduces overfitting.

**4.1.4 Hyperparameter Tuning and Cross-Validation:**

Enhancing the input feature space through feature selection based on the ChWO algorithm was the optimization strategy's main objective. By making sure that only the most relevant features were included for classification, this technique successfully decreased feature redundancy, increased class separability, and improved overall model performance.

- **Batch Size and Learning Rate:** To balance memory limitations with training stability, we chose widely used empirically validated settings a fixed learning rate of 1e-4 and a batch size of 32.
- **Optimizer:** The Adam optimizer was chosen because of its demonstrated convergence efficiency in deep learning problems, and it has a weight decay of 1e-5.
- **Training Epochs:** Up to 100 training epochs were used to give the models enough time to converge without being overfit.

**4.2 Comparative Assessment**

The comparative assessment is devised using two various datasets like D1 and D2 with the existing Thorax disease detection methods like ThoraX-PriorNet [21], CXray-EffDet [22], Attention-based CNN [23], and Swin Transformer [26]. The definition for the assessment measures is portrayed in Table 2.

**Table 2:** Definition for Assessment Measures

| **Measures** | **Formula** | **Symbol Definition** |
| --- | --- | --- |
| **Accuracy** | $\frac{TP+TN}{TP+TN+FP+FN}$ | $TP$True Positive  $TN$True Negative  $FP$False Positive  $FN$False Negative |
| **Precision** | $\frac{TP}{TP+FP}$ |  |
| **Recall** |  |  |
| **F-Measure** | $2\times\frac{\Pr ecision\times\mathrm{Re}call}{\Pr ecision+\mathrm{Re}call}$ |  |
| **MCC** | $MCC=\frac{TP\cdot TN-FP\cdot FN}{\sqrt{\left(TP+FP\right)\left(TP+FN\right)\left(TN+FP\right)\left(TN+FN\right)}}$ |  |
| **MAE** |  | $\hat{y}_{i}$-predicted value  $y_{i}$-actual value  $n$-number of observation |

**4.2.1 Assessment Using Dataset 1**

The assessment of Thorax disease classification method using the D1 dataset is evaluated based on training data and is elaborated in this section.

The accuracy-based performance evaluation of several models at varying training percentages is displayed in Figure 6. Accuracy defines the proportion of correct predictions made by the model out of the total samples. The accuracy assessment of Thorax disease classification methods is portrayed in Figure 6 (a). Here, the EnAE is key to extracting critical attributes using stacked auto-encoders combined with an attention module, EnAE extracts important features, removing noise and irrelevant data. In addition, IMSTrans leverages advanced attention mechanisms and hierarchical feature extraction, allowing it to process image patches efficiently. With values ranging from 0.878 at 50% training to 0.964 at 90%, the suggested model achieves the highest accuracy and routinely outperforms alternative approaches. Thus, the proposed EnAE+ChWO+IMSTrans model captures both local and global patterns in the X-rays, which leads to more accurate predictions compared to existing approaches. The precision analysis portrayed in Figure 6 (b). Here, the ChWO algorithm utilized to select the most significant features after extraction. By discarding less relevant attributes, the model is streamlined to focus on features that are strongly correlated with specific thoracic diseases, reducing the risk of making incorrect classifications. The model's highly accurate performance in predicting positives is demonstrated by the proposed method's highest precision of 0.977 at 90% training percentage. Thus, the incorrect classifications are eliminated by the proposed model that leads to higher precision by the proposed EnAE+ChWO+IMSTrans model. The recall-based analysis is demonstrated in Figure 6 (c) The proposed method achieves higher recall due to the balanced data and efficient feature extraction modules. By augmenting the minority class samples, the model becomes better at identifying true cases of Thorax diseases that assist in improving its recall. In addition, the EnAE captures finer and more complex features, ensuring that the model can detect even minor indicators of thoracic diseases, reducing false negatives. With the highest recall of 0.9845 at 90% training percentage, the suggested technique accurately identify the majority of relevant instances.

|  |  |
| --- | --- |
| **(a)** | **(b)** |
|  |  |
| **(c)** | **(d)** |
|  |  |
| **(e)** | **(f)** |

**Figure 6:** Assessment Using Dataset 1: (a) Accuracy, (b) Precision, (c) Recall, (d) F-Measure, (e) MCC and (f) MAE

The F1-score-based analysis is demonstrated in Figure 6 (d) The EnAE’s ability to extract significant features and IMSTrans’s powerful classification ensure that both precision and recall are maximized. It indicates that the proposed model helps accomplish both sensitive in detecting diseases (high recall) and specificity in making correct predictions (high precision). The maximum F1-Score of 0.964 is obtained by the proposed method at 90% training percentage, demonstrating an effective trade-off between recall and precision. Matthews Correlation Coefficient (MCC) is measured to analyze the robustness of the Thorax disease for both positive and negative classes. The MCC-based analysis is demonstrated in Figure 6 (e). The use of data augmentation helps reduce the bias toward majority classes that results in a more balanced classification. It leads to lower false positive and false negative rates, which contribute to a higher MCC score by the proposed EnAE+ChWO+IMSTrans model. The proposed method achieves the maximum MCC of 0.9647 at 90%, indicating that it is the most dependable and balanced model. The MAE-based analysis is demonstrated in Figure 6 (f). EnAE minimizes reconstruction error when extracting features, which ensures that the latent representations of the X-rays are highly accurate. It reduces the overall error in classifying the Thorax disease. The careful selection of features helps minimize prediction errors. By ensuring that only relevant features are used, the model avoids making large errors, which keeps the MAE low. The proposed method has the lowest MAE of 0.184 at 90%, meaning it predicts with the least amount of error. Table 3 shows the Assessment Using Dataset 1

**Table 3.** Assessment Using Dataset 1

| Methods/ Training  Percentage | 50 | 60 | 70 | 80 | 90 |
| --- | --- | --- | --- | --- | --- |
| Accuracy | | | | | |
| ThoraX-PriorNet | 0.806 | 0.812 | 0.843 | 0.884 | 0.887 |
| CXray-EffDet | 0.813 | 0.822 | 0.835 | 0.905 | 0.912 |
| Attention based CNN | 0.836 | 0.834 | 0.846 | 0.891 | 0.928 |
| Swin Transformer | 0.842 | 0.844 | 0.851 | 0.913 | 0.945 |
| **Proposed** | **0.878** | **0.896** | **0.909** | **0.92** | **0.964** |
| **Precision** | | | | | |
| ThoraX-PriorNet | 0.8265 | 0.8705 | 0.876 | 0.8942 | 0.9115 |
| CXray-EffDet | 0.8452 | 0.872 | 0.8885 | 0.94 | 0.944 |
| Attention based CNN | 0.891 | 0.917 | 0.9255 | 0.929 | 0.958 |
| Swin Transformer | 0.8737 | 0.8794 | 0.9078 | 0.9239 | 0.9534 |
| **Proposed** | **0.896** | **0.903** | **0.931** | **0.9515** | **0.977** |
| **Recall** | | | | | |
| ThoraX-PriorNet | 0.778 | 0.7455 | 0.806 | 0.8825 | 0.8715 |
| CXray-EffDet | 0.7925 | 0.77 | 0.7865 | 0.867 | 0.874 |
| Attention based CNN | 0.7855 | 0.772 | 0.785 | 0.8915 | 0.931 |
| Swin Transformer | 0.8256 | 0.8597 | 0.8662 | 0.8926 | 0.9578 |
| **Proposed** | **0.855** | **0.8815** | **0.8935** | **0.921** | **0.9845** |
| **F1-Score** | | | | | |
| ThoraX-PriorNet | 0.8023 | 0.8041 | 0.8392 | 0.8885 | 0.892 |
| CXray-EffDet | 0.8178 | 0.8195 | 0.838 | 0.9045 | 0.9118 |
| Attention based CNN | 0.8367 | 0.8385 | 0.8502 | 0.8955 | 0.9337 |
| Swin Transformer | 0.8481 | 0.8712 | 0.8842 | 0.9089 | 0.9423 |
| **Proposed** | **0.8768** | **0.894** | **0.9125** | **0.9385** | **0.964** |
| **MCC** | | | | | |
| ThoraX-PriorNet | 0.7793 | 0.7476 | 0.8102 | 0.8827 | 0.8743 |
| CXray-EffDet | 0.7924 | 0.7712 | 0.7888 | 0.8697 | 0.8771 |
| Attention based CNN | 0.8074 | 0.8128 | 0.8887 | 0.8914 | 0.9428 |
| Swin Transformer | 0.8355 | 0.8605 | 0.8686 | 0.8976 | 0.9368 |
| **Proposed** | **0.8558** | **0.8819** | **0.8971** | **0.9242** | **0.9647** |
| **MAE** | | | | | |
| ThoraX-PriorNet | 0.2585 | 0.2354 | 0.2218 | 0.2182 | 0.2148 |
| CXray-EffDet | 0.2473 | 0.2326 | 0.2237 | 0.2161 | 0.2106 |
| Attention based CNN | 0.2434 | 0.231 | 0.2169 | 0.2164 | 0.2049 |
| Swin Transformer | 0.2401 | 0.2226 | 0.2137 | 0.2129 | 0.2001 |
| **Proposed** | **0.233** | **0.2143** | **0.2048** | **0.2017** | **0.184** |

**4.2.1 Assessment Using Dataset 2**

The assessment of Thorax disease classification method using the D2 dataset is evaluated based on training data and is elaborated in this section.

|  |  |
| --- | --- |
| **(a)** | **(b)** |
|  |  |
| **(c)** | **(d)** |
|  |  |
| **(e)** | **(f)** |

**Figure 7:** Assessment Using Dataset 2: (a) Accuracy, (b) Precision, (c) Recall, (d) F-Measure, (e) MCC and (f) MAE

Figure 7 analyzes the performance of various Thorax disease classification techniques on the D2 dataset, comparing their efficacy at different training data proportions, ranging from 50% to 90%. The accuracy is shown in 7(a), where EnaE+CHWO+IMSTrans continuously attains the best accuracy, hitting roughly 0.96 at 90% training data. Likewise, in 7 (b) Precision, EnaE+CHWO+IMSTrans is in the lead with approximately 0.95 at 90%. In 7(c), the Recall exhibits a comparable pattern, with EnaE+CHWO+IMSTrans reaching roughly 0.94. The F-Measure in 7(d), which strikes a balance between recall and precision, likewise peaks for EnaE+CHWO+IMSTrans at about 0.94. Figure 7 (e) shows that the MCC scores for all models generally improve as the training percentage rises from 50% to 90%. In particular, proposed EnAe+CHWO+IMSTrans attains the greatest MCC with 90% training data, at roughly 0.96.  At the maximum training , ThoraX-PriorNet has the lowest MCC, circling 0.86. This suggests that, particularly when trained on more data, EnAe+CHWO+IMSTrans have the greatest overall classification performance. Figure (f) shows that the MAE for all models tends to drop as the training rises. EnAe+CHWO+IMSTrans shows the lowest MAE at the 90% training level, which is at 0.04, indicating the smallest average magnitude of errors in its predictions. At 90% training data, ThoraX-PriorNet has the greatest MAE, approximately 0.09, indicating higher average prediction errors. This suggests that, when trained with a larger dataset, EnAe+CHWO+IMSTrans produces the most accurate numerical predictions out of all the models that were compared. Table 4 shows the assessment using Dataset 2.

**Table 4.** Assessment Using Dataset 2

| Methods/ Training  Percentage | 50 | 60 | 70 | 80 | 90 |
| --- | --- | --- | --- | --- | --- |
| Accuracy | | | | | |
| ThoraX-PriorNet | 0.823 | 0.828 | 0.853 | 0.903 | 0.91 |
| CXray-EffDet | 0.804 | 0.841 | 0.868 | 0.901 | 0.927 |
| Attention based CNN | 0.845 | 0.85 | 0.902 | 0.913 | 0.946 |
| Swin Transformer | 0.862 | 0.861 | 0.887 | 0.926 | 0.959 |
| **Proposed** | **0.893** | **0.908** | **0.926** | **0.944** | **0.986** |
| **Precision** | | | | | |
| ThoraX-PriorNet | 0.817 | 0.859 | 0.872 | 0.892 | 0.918 |
| CXray-EffDet | 0.841 | 0.885 | 0.894 | 0.909 | 0.936 |
| Attention based CNN | 0.858 | 0.891 | 0.913 | 0.942 | 0.955 |
| Swin Transformer | 0.876 | 0.885 | 0.931 | 0.926 | 0.962 |
| **Proposed** | **0.899** | **0.911** | **0.941** | **0.963** | **0.985** |
| **Recall** | | | | | |
| ThoraX-PriorNet | 0.784 | 0.742 | 0.789 | 0.862 | 0.877 |
| CXray-EffDet | 0.797 | 0.788 | 0.804 | 0.856 | 0.879 |
| Attention based CNN | 0.823 | 0.838 | 0.841 | 0.903 | 0.9 |
| Swin Transformer | 0.876 | 0.899 | 0.91 | 0.897 | 0.926 |
| **Proposed** | **0.883** | **0.902** | **0.932** | **0.946** | **0.985** |
| **F1-Score** | | | | | |
| ThoraX-PriorNet | 0.801 | 0.81 | 0.843 | 0.876 | 0.89 |
| CXray-EffDet | 0.822 | 0.834 | 0.834 | 0.887 | 0.913 |
| Attention based CNN | 0.846 | 0.849 | 0.867 | 0.9 | 0.942 |
| Swin Transformer | 0.833 | 0.851 | 0.873 | 0.92 | 0.94 |
| **Proposed** | **0.872** | **0.897** | **0.917** | **0.946** | **0.993** |
| **MCC** | | | | | |
| ThoraX-PriorNet | 0.779 | 0.762 | 0.825 | 0.874 | 0.877 |
| CXray-EffDet | 0.801 | 0.797 | 0.814 | 0.859 | 0.9 |
| Attention based CNN | 0.829 | 0.831 | 0.894 | 0.903 | 0.942 |
| Swin Transformer | 0.823 | 0.848 | 0.883 | 0.911 | 0.942 |
| **Proposed** | **0.861** | **0.887** | **0.903** | **0.935** | **0.978** |
| **MAE** | | | | | |
| ThoraX-PriorNet | 0.177 | 0.172 | 0.147 | 0.097 | 0.09 |
| CXray-EffDet | 0.196 | 0.159 | 0.132 | 0.099 | 0.073 |
| Attention based CNN | 0.155 | 0.15 | 0.098 | 0.087 | 0.054 |
| Swin Transformer | 0.138 | 0.139 | 0.113 | 0.074 | 0.041 |
| **Proposed** | **0.107** | **0.092** | **0.074** | **0.056** | **0.014** |

**4.3 Accuracy-Loss:** Accuracy-loss analysis compares the model's accuracy and loss during both training and testing phases to assess the performance of the model and determine if it's generalizing well to unseen data.

The accuracy and loss trends for Dataset 1 to classify thorax disease over 100 epochs are displayed in Figure 8. Over 100 epochs, the accuracy trends are shown in Figure 8(a). The accuracy of training rises quickly, peaking at about 95% around epoch 30 before plateauing. The trajectory of the testing accuracy is similar, with modest swings after a slightly lower peak at 94%. This shows that the model is capable of learning and generalizing to new data with reasonable accuracy. In Figure 8(b), the loss curves are shown. Over time, the training loss decreases from roughly 0.7 to less than 0.2. After a brief period of volatility, the testing loss likewise drops dramatically, stabilizing at about 0.2. It is clear from the rather narrow difference between training and testing loss that the model is not significantly overfitting the training set.

|  |  |
| --- | --- |
| (a) | (b) |

**Figure 8.** Accuracy-Loss Analysis for Dataset 1

**Figure 9.** Accuracy-Loss Analysis for Dataset 2

The accuracy and loss trends for Dataset 2 to classify thorax disease over 100 epochs are displayed in Figure 9. Minor overfitting is suggested by the testing loss, which drops to a minimum of 0.21 before slightly increasing to 0.22, while the training loss gradually reduces from roughly 0.7 to 0.19. On the other hand, the black training accuracy quickly increases from 0.5 to a plateau at 0.97. Also improving is the testing accuracy, which peaks at about 0.95 and stays steady. Although there is some overfitting in later epochs, the model's overall high testing accuracy of almost 95% shows that learning is effective.

**4.4 Confusion Matrix:** The confusion matrix representation of the proposed EnAE+ChWO+IMSTrans model using dataset 1 and dataset 2 is portrayed in Figure 10, which illustrates the correct classification made by the proposed method.

|  |  |
| --- | --- |
| (a) | (b) |

**Figure 10**: Confusion Matrix for (a) Dataset 1 and (b) Dataset 2

Figure 10 (a) shows a 14x14 matrix for thorax disease classification. For a number of classes, including class 0 (82), class 2 (71), and class 4 (107), the diagonal displays high correct classifications. Misclassifications sometimes occur, though; for example, 37 cases of class 1 were incorrectly classified as class 0. The three categories of fibrosis, normal, and pneumonia are simplified in Figure 10 (b). With 250 pneumonia cases correctly predicted, 201 normal cases correctly classified, and 573 fibrosis patients correctly recognized, the matrix shows strong results. Three cases of pneumonia were misclassified as fibrosis, while two cases of normal were misclassified as fibrosis. At 0.986, this classification's overall accuracy score on Dataset 2 is remarkably high. The model's accuracy in classifying thoracic disorders is clearly demonstrated by these matrices, with Dataset 2 exhibiting especially strong findings.

**4.5 AUC Analysis:** The AUC analysis of Dataset 1 is portrayed in Figure 11. The area under the ROC curve is ranging from 0 to 1. A higher AUC value by EnAE+ChWO+IMSTrans model indicates better performance in distinguishing between classes. The analysis of Dataset 2's Area Under the Curve (AUC) is shown in Figure 12. Various model's performances are evaluated in 12 (a), and the model with the greatest AUC, EnAE+ClWO+IMSTrans, shows superior classification abilities. The AUC curves for several classes (0, 1, and 2) are shown in 12(b). All of these classes attain unusually high AUC scores of 0.99, indicating superior discrimination ability across all Dataset 2 categories.

|  |  |
| --- | --- |
| (a) | (b) |

**Figure 11.** AUC Analysis for Dataset 1

|  |  |
| --- | --- |
| (a) | (b) |

**Figure 12.** AUC Analysis for Dataset 2

**4.6 Convergence Analysis:** Convergence analysis evaluates how quickly and effectively an optimization algorithm minimizes the loss function, ensuring that the model reaches the global or near-global minimum efficiently. The convergence analysis is demonstrated in Figure 13. The inclusion of chaotic mapping assist the proposed ChWO algorithm to converge faster compared to existing whale optimization algorithm.

**Figure 13.** Convergence Analysis

**4.7 Comparative Discussion**

The comparative discussion for Dataset 1 and Dataset 2 based on the best case with 90% of training data and 10% of testing data is portrayed in Table 5. The proposed EnAE+ChWO+IMSTrans approach performs better across all assessment measures, according to a comparative analysis of Thorax disease categorization on Datasets 1 and 2. The proposed approach outperformed ThoraX-PriorNet, CXray-EffDet, Attention-based CNN, and Swin Transformer by 7.99%, 5.39%, 3.73%, and 1.97%, respectively, and obtained the highest accuracy (0.964) for Dataset 1. Likewise, the proposed technique outperformed Swin Transformer, the next best method, by 2.81% for Dataset 2, achieving an accuracy of 0.986. Throughout both datasets, the proposed approach continuously produced superior outcomes in terms of accuracy, recall, F-score, and MCC. Its robustness in detecting true positives is noteworthy, as evidenced by the recall on Datasets 1 (0.9845) and 2 (0.985). Additionally, the model's Mean Absolute Error (MAE) was the lowest, particularly on Dataset 2, at 0.014, which is far lower than all baseline techniques. These findings highlight the improved generality and efficacy of the proposed method in classifying Thorax disease, particularly when 90% of the data is used for training and 10% is used for testing. Overall, the two dataset's consistent superiority highlights its appropriateness for dependable clinical use in medical image classification tasks.

**Table 5.** Comparative Analysis for Dataset 1 and Dataset 2

| Methods/ Metrics | Accuracy | Precision | Recall | F-Score | MCC | MAE |
| --- | --- | --- | --- | --- | --- | --- |
| Dataset 1 | | | | | | |
| ThoraX-PriorNet | 0.887 | 0.9115 | 0.8715 | 0.892 | 0.8743 | 0.2148 |
| CXray-EffDet | 0.912 | 0.944 | 0.874 | 0.9118 | 0.8771 | 0.2106 |
| Attention based CNN | 0.928 | 0.958 | 0.931 | 0.9337 | 0.9428 | 0.2049 |
| Swin Transformer | 0.945 | 0.9534 | 0.9578 | 0.9423 | 0.9368 | 0.2001 |
| **Proposed** | **0.964** | **0.977** | **0.9845** | **0.964** | **0.9647** | **0.184** |
| Dataset 2 | | | | | | |
| ThoraX-PriorNet | 0.91 | 0.918 | 0.877 | 0.89 | 0.877 | 0.09 |
| CXray-EffDet | 0.927 | 0.936 | 0.879 | 0.913 | 0.9 | 0.073 |
| Attention based CNN | 0.946 | 0.955 | 0.9 | 0.942 | 0.942 | 0.054 |
| Swin Transformer | 0.959 | 0.962 | 0.926 | 0.94 | 0.942 | 0.041 |
| **Proposed** | **0.986** | **0.985** | **0.985** | **0.993** | **0.978** | **0.014** |

Here, the comparative analysis portrays the superiority of the proposed model compared to the existing methods.

**Table 6.** Comparative Analysis of proposed and state-of-the-art methods

| **Dataset 1** | | | |
| --- | --- | --- | --- |
| Methods/ Metrics | Accuracy | Precision | Recall |
| CX-Ultranet [30] | 0.844 | 0.857 | 0.835 |
| DenseNet121 [31] | 0.861 | 0.874 | 0.849 |
| CycleGAN [32] | 0.933 | 0.947 | 0.921 |
| **Proposed (90%)** | **0.964** | **0.977** | **0.9845** |
| **Dataset 2** | | | |
| CX-Ultranet | 0.842 | 0.832 | 0.819 |
| DenseNet121 | 0.867 | 0.869 | 0.842 |
| CycleGAN | 0.949 | 0.956 | 0.942 |
| **Proposed (90%)** | **0.986** | **0.985** | **0.985** |

A comparative analysis of proposed and state-of-the-art methods for classifying thoracic diseases across two datasets is shown in Table 6. The suggested approach achieves the highest accuracy, precision, and recall at 90%, outperforming all others. It considerably outperforms CycleGAN with 93.3% accuracy, 94.7% precision, and 92.1% recall on Dataset 1, achieving 96.4% accuracy, 97.7% precision, and 98.45% recall. Likewise, the suggested approach attains 98.6% accuracy, 98.5% precision, and 98.5% recall in Dataset 2. On the other hand, DenseNet121 and CX-Ultranet perform moderately, demonstrating the superior efficacy of the proposed model in classifying thoracic diseases.

**Reviewer 2’s comments to author:**

Strengths of the Paper:

1. The proposed framework effectively addresses class imbalance through data augmentation, enhancing model generalization.

2. The combination of stacked auto-encoder architecture with an attention module in EnAE improves feature representation and classification accuracy.

3. The utilization of the Chaotic Whale Optimization Algorithm refines feature selection, ensuring optimal attributes are used for classification.

4. The IMSTrans model efficiently processes high-dimensional medical image data, leading to improved classification performance.

5. The experimental results indicate the proposed method achieves high performance metrics, demonstrating its reliability and efficiency in thorax disease classification.

Main Comments for Improvement:

1. Recent References: The paper should include references from 2024, ensuring the literature review is up-to-date and reflects the latest advancements in thoracic disease classification and deep learning applications.

**Response:** We thank the reviewer for this valuable suggestion. In response, we have updated the literature review section by replacing outdated references with recent and relevant studies from 2024. These additions better reflect the current advancements in thoracic disease classification and deep learning. Corresponding updates have also been made in the reference section to ensure consistency. The changes are highlighted red colour in the revised research manuscript in section 2. The changes are described as follows;

A CX-Ultranet was designed by [30] for classify and diagnose 13 distinct thoracic illnesses from plain radiography pictures. A multiclass cross-entropy loss function was used within a compound scaling structure, and EfficientNet was used as the baseline model. In order to create reduction cells and add more skip connections, channel shuf-fling was applied at different points along the network. Together, the loss function algorithm and Adam optimizers stabilize the model and enable ongoing learning from fresh data over time.

A transfer learning method for categorizing radiological abnormalities and respiratory conditions from chest X-rays was designed by [31]. There were 752 X-ray photos from the University Clinical Center of Kragujevac and 191,660 public images in the dataset. Infection, pleural thickening, atelectasis, cardiomegaly, tuberculosis, malignancies, non-viral and COVID-19 pneumonia, and healthy cases were among the disorders it covers. This research, in contrast to others, defines up to 18 illness groups and separates healthy from diseased instances. DenseNet121 with CheXNeXt weights and additional layers for fine-tuning are used in the procedure.

CycleGAN-based preprocessing approach for enhanced lung disease classification in ChestX-Ray14 was designed by [32] that starts with identifying images with artifacts and then uses a CycleGAN model to produce sharper images in order to lessen the noise effect of the artifacts. Channel and spatial attention methods are used by the DenseNet-121 model, which is used for classification, to target certain areas of the image. The model was also updated to incorporate additional data from the dataset, specifically clinical features. Table 1 shows the analysis of recent research work.

**References**

30.	Kabiraj, A., Meena, T., Reddy, P.B. and Roy, S., 2024. Multiple thoracic diseases detection from X-rays using CX-Ultranet. Health and Technology, 14(2), pp.291-303.

31.	Geroski, T., Pavić, O., Dašić, L., Milovanović, D., Petrović, M. and Filipović, N., 2024. SoftLungX: leveraging transfer learning with convolutional neural networks for accurate respiratory disease classification in chest X-ray images. Journal of Big Data, 11(1), p.146.

32.	Chehade, A.H., Abdallah, N., Marion, J.M., Hatt, M., Oueidat, M. and Chauvet, P., 2025. Advancing chest X-ray diagnostics: A novel CycleGAN-based preprocessing approach for enhanced lung disease classifi-cation in ChestX-Ray14. Computer Methods and Programs in Biomedicine, 259, p.108518.

2. Comparison with State-of-the-Art: The results should be compared with recent state-of-the-art methods. Additionally, citations should be included in the comparative analysis to support claims of superior performance.

**Response:** We thank the reviewer for the valuable suggestion. In the revised manuscript, we have included a comprehensive comparison with recent state-of-the-art methods. This comparison highlights the performance of the proposed approach relative to other leading techniques across standard benchmarks. We have also included appropriate citations to support the reported results and ensure fair and accurate comparison. These additions strengthen the validity and demonstrate the superiority of proposed method. The changes are highlighted red colour in the revised research manuscript in section 4.7. The changes are described as follows;

**Table 6.** Comparative Analysis of proposed and state-of-the-art methods

| **Dataset 1** | | | |
| --- | --- | --- | --- |
| Methods/ Metrics | Accuracy | Precision | Recall |
| CX-Ultranet [30] | 0.844 | 0.857 | 0.835 |
| DenseNet121 [31] | 0.861 | 0.874 | 0.849 |
| CycleGAN [32] | 0.933 | 0.947 | 0.921 |
| **Proposed (90%)** | **0.964** | **0.977** | **0.9845** |
| **Dataset 2** | | | |
| CX-Ultranet | 0.842 | 0.832 | 0.819 |
| DenseNet121 | 0.867 | 0.869 | 0.842 |
| CycleGAN | 0.949 | 0.956 | 0.942 |
| **Proposed (90%)** | **0.986** | **0.985** | **0.985** |

A comparative analysis of proposed and state-of-the-art methods for classifying thoracic diseases across two datasets is shown in Table 6. The suggested approach achieves the highest accuracy, precision, and recall at 90%, outperforming all others. It considerably outperforms CycleGAN with 93.3% accuracy, 94.7% precision, and 92.1% recall on Dataset 1, achieving 96.4% accuracy, 97.7% precision, and 98.45% recall. Likewise, the suggested approach attains 98.6% accuracy, 98.5% precision, and 98.5% recall in Dataset 2. On the other hand, DenseNet121 and CX-Ultranet perform moderately, demonstrating the superior efficacy of the proposed model in classifying thoracic diseases.

**References**

30.	Kabiraj, A., Meena, T., Reddy, P.B. and Roy, S., 2024. Multiple thoracic diseases detection from X-rays using CX-Ultranet. Health and Technology, 14(2), pp.291-303.

31.	Geroski, T., Pavić, O., Dašić, L., Milovanović, D., Petrović, M. and Filipović, N., 2024. SoftLungX: leveraging transfer learning with convolutional neural networks for accurate respiratory disease classification in chest X-ray images. Journal of Big Data, 11(1), p.146.

32.	Chehade, A.H., Abdallah, N., Marion, J.M., Hatt, M., Oueidat, M. and Chauvet, P., 2025. Advancing chest X-ray diagnostics: A novel CycleGAN-based preprocessing approach for enhanced lung disease classifi-cation in ChestX-Ray14. Computer Methods and Programs in Biomedicine, 259, p.108518.

3. Acronyms Usage: The full form of each acronym should be provided only at its first mention in the text. Subsequent mentions should use only the acronym to maintain readability and consistency.

**Response:** Thank you for your valuable feedback regarding the use of acronyms in the manuscript. We appreciate your suggestion and have revised the text accordingly. In response to reviewer comments, the full form of each acronym is now provided at its first mention, followed by the use of the acronym throughout the remainder of the manuscript. This approach ensures clarity while maintaining readability and consistency.

4. Problem Statement References: The problem statement section should be supported with appropriate references to establish the significance and relevance of the study.

**Response:** Thank you for your valuable feedback. We have updated the Problem Statement section of the manuscript to include appropriate references that support the significance and relevance of the study. These references provide a stronger foundation for the study’s objectives and highlight its importance in the broader context of current research. The changes are highlighted red colour in the revised research manuscript in section 2.1. The changes are described as follows;

**2.1 Problem Statement**

Deep learning for thoracic disease classification and localization has advanced, however there are still a number of important research gaps that highlight the importance and applicability of more study in this area. Numerous current models, like the ResNet-based Triplet-Attention model [29] and ThoraX-PriorNet [21], mostly rely on anatomical priors and attention mechanisms. These methods enhance computing complexity and improve accuracy, but they also introduce dependence on the quality of earlier predictions. Similar to this, models like CXray-EffDet [22] and CX-Ultranet [30] use lightweight backbones like EfficientNet to promote computational efficiency, but they still struggle to handle anatomical changes and fluctuating image quality. Although Vision Transformer-based methods [24] provide better multi-label classification, their high computing requirements limit their scalability. Additionally, the majority of research suffers from inadequate generalizability across various clinical contexts, lack of interpretability [25], and dataset imbalance [23]. Despite recent attempts to use CycleGANs to enhance preprocessing and integrate clinical metadata [32], multimodal data integration is still not completely recognized. Furthermore, transfer learning techniques [31] have the potential to benefit from massive data sets, but fine-tuning for domain-specific subtleties remains a difficult task. Thus, the goal is to create a robust system that improves diagnostic accuracy, reduces false positives and negatives, and supports radiologists in making faster, more accurate decisions, ultimately improving patient outcomes. This research is motivated by the difficulties of correctly identifying thoracic disease from chest X-ray scans, which frequently entail complicated anatomical variances and imbalanced datasets. A novel EnAE with an attention mechanism for robust feature extraction, sophisticated data augmentation techniques to reduce class imbalance, and the ChWO algorithm for optimal feature selection are all used in the proposed approach to address these problems. Finally, the IMSTrans model facilitates accurate classification of thoracic diseases, enhancing precision and facilitating improved clinical decision-making.

5. Proposed Methodology References: The methodology should be reinforced with citations, particularly when discussing techniques such as EnAE, the Chaotic Whale Optimization Algorithm, SWIN, and IMSTrans, to establish credibility and provide background for the employed techniques.

**Response:** We appreciate the reviewer’s valuable feedback. In the revised manuscript, we have ensured that all key techniques mentioned in the methodology section including Enhanced Autoencoder (EnAE), the Chaotic Whale Optimization Algorithm (CWOA), SWIN Transformer, and IMSTrans are properly cited with relevant and recent references. These citations provide both credibility and a solid background for the employed techniques. The changes are highlighted red colour in the revised research manuscript in section 3 and references are given below. The changes are described as follows;

**References**

26.	Li, W., Zou, X., Zhang, J., Hu, M., Chen, G. and Su, S., 2025. R predicting lung cancer bone metastasis using CT and pathological imaging with a Swin Transformer model. Journal of Bone Oncology, p.100681.

33. Yuan, X., Qi, S. and Wang, Y., 2020. Stacked enhanced auto-encoder for data-driven soft sensing of quality variable. IEEE Transactions on Instrumentation and Measurement, 69(10), pp.7953-7961.

34. Cui, L., Jing, X., Wang, Y., Huan, Y., Xu, Y. and Zhang, Q., 2022. Improved swin transformer-based se-mantic segmentation of postearthquake dense buildings in urban areas using remote sensing images. IEEE Journal of Selected Topics in Applied Earth Observations and Remote Sensing, 16, pp.369-385.

36. Sayed, G.I., Darwish, A. and Hassanien, A.E., 2018. A new chaotic whale optimization algorithm for fea-tures selection. Journal of classification, 35, pp.300-344.

6. Data Distribution Analysis: The paper does not mention the data distributions before and after augmentation. It is essential to provide details on the original dataset distribution and how augmentation affects class balance and feature diversity.

**Response:** Thank you for your comment. We had provided the details of the data distributions both before and after augmentation in the manuscript. The original dataset distribution and the impact of augmentation on class balance and feature diversity were discussed to demonstrate how augmentation improved the representativeness and robustness of the dataset. The changes are highlighted red colour in the revised research manuscript in section 4.1. The changes are described as follows;

**4.1 Dataset Description**

The datasets like ChestX-Ray dataset [27] and Lung Disease Dataset [35] are utilized for the evaluation of the proposed EnAE+ChWO+IMSTrans-based Thorax disease classification.

**4.1.1 Original Data Distribution:**

**Dataset 1: ChestX-Ray dataset**

- The dataset consists of 8,274 chest X-ray images across 14 thoracic disease categories:
- Atelectasis, Cardiomegaly, Effusion, Infiltration, Mass, Nodule, Pneumonia, Pneumothorax, Consolidation, Edema, Emphysema, Fibrosis, Pleural Thickening, and Hernia.
- These images are annotated with one or more disease labels, and the dataset is inherently multi-label and imbalanced. For instance, diseases such as Infiltration and Effusion are more frequent, while Hernia and Fibrosis are rare.
- This imbalance was initially visualized through class-wise frequency plots to better understand the skewness in data distribution.

**Dataset 2: Lung Disease Dataset**

- This dataset contains 4,260 high-resolution chest X-ray images organized into three classes:
- Normal Lungs (no abnormalities),
- Fibrosis Lungs and
- Pneumonia Lungs.
- The data also exhibits imbalance, with Normal cases being more prevalent compared to Fibrosis and Pneumonia.

**4.1.2 Data Distributions and Preprocessing Steps**

**Dataset 1: ChestX-Ray dataset**

- **Source**: Publicly available NIH ChestX-ray14 dataset.
- **Subset Size Used**: 8,274 chest X-ray images.
- **Classes** (14 in total):
  Atelectasis, Cardiomegaly, Effusion, Infiltration, Mass, Nodule, Pneumonia, Pneumothorax, Consolidation, Edema, Emphysema, Fibrosis, Pleural Thickening, Hernia.
- **Nature**: Multi-label, class-imbalanced dataset.
  Infiltration and Effusion are overrepresented, while Hernia and Fibrosis are rare.
- **Label Handling**: The "Finding Labels" column is parsed to assign binary multi-label vectors for each class.
- **Preprocessing:**
  - Images with missing or unreadable files were removed.
  - Images resized to 256×256, followed by random crop (224×224).
  - Pixel normalization with ImageNet statistics.
  - Missing or null labels were excluded.
- **Data Augmentation**:
  - Random horizontal and vertical flips (p=0.5).
  - Random rotations (up to ±30 degrees).
  - Augmentation increases feature variability but does not alter label distribution.

**Dataset 2: Lung Disease Dataset**

- **Source**: dataset containing **4,260 images**.
- **Classes**: Normal, Pneumonia, and Fibrosis.
- **Nature**: Multi-label, class-imbalanced dataset.
- **Preprocessing:**
  - Images resized and normalized using the same procedure as Dataset 1 to ensure consistency.
  - Verified for label completeness and removed any ambiguous or mislabeled samples.

**4.1.3 Data Augmentation and Impact:**

To address the data imbalance and enhance model generalization, applied a robust augmentation pipeline during training that includes:

- Spatial transformations (resizing, random cropping),
- Geometric variations (horizontal/vertical flipping, random rotations),
- Normalization to standardize image intensity.

These augmentations are applied dynamically at each training epoch. While the underlying image count remains unchanged, the model is exposed to diverse representations of each sample, especially from minority classes. This synthetically balances the training data, increases intra-class variance, and reduces overfitting.

7. Method Limitations and Future Work: The paper should discuss the limitations of the proposed method, such as computational complexity, dependency on dataset quality, or generalizability to other medical imaging modalities. Additionally, future research directions should be outlined.

**Response:** We appreciate the reviewer’s valuable suggestion. In response, we have added a dedicated section in the revised manuscript discussing the limitations of our proposed method. Specifically, we now address potential computational complexity, the method’s dependency on the quality and diversity of the training dataset, and challenges related to generalizability across different medical imaging modalities. Furthermore, we have outlined several directions for future research. The changes are highlighted red colour in the revised research manuscript in section 5.1. The changes are described as follows;

**5.1 Limitations and Future Work**

The proposed method faces certain limitations even if it shows better classification performance when employing the Chaotic Whale Optimization Algorithm for feature selection and the EnAE with attention mechanisms. First and foremost, it is computationally demanding, particularly during the training and feature extraction stages, using significant resources that could impede its use in environments with limited resources, such remote hospitals or mobile diagnostic units. Furthermore, the quality and representativeness of the Chest X-ray dataset used are critical to the model's performance; even with data augmentation efforts, imbalances and rare illness classes continue to be problems. There is currently no test for generalizability to other medical imaging modalities, which restricts wider clinical use.

Thus, Future research should carry out comprehensive clinical validation tests with radiologists in order to overcome the uncertainties around the model's applicability in actual clinical situations. In order to assess the model's interpretability, dependability, and practical utility in routine diagnostics, such investigations are crucial. Refinements can be guided by collaborations with doctors to better address clinical demands. Trust and usability will be improved by incorporating explainability elements. Furthermore, deployment requires optimizing computing efficiency, particularly in environments with restricted resources. It will be easier to move from a research prototype to a clinically useful tool for diagnosing thorax disease if the dataset is expanded to include multi-institutional and multi-modal imaging data.

**Reviewer 3’s comments to author:**

Thank you for the opportunity to review " Improved Swin Transformer based Thorax Disease Classification with Optimal Feature Selection using Chest X-ray”.

While the topic of the submitted article of great importance, minor concerns exist regarding the current version of the manuscript.

1. The introduction needs a clearer articulation of the research gap and objectives.

**Response:** We thank the reviewer for this valuable suggestion. In response, we have revised the introduction and clearly articulate the specific research gap our study addresses. We have also explicitly stated the objectives of the study towards the end of the introduction to provide better clarity and focus. These changes help to better position our work within the existing literature and highlight its contributions. The changes are highlighted red colour in the revised research manuscript in section 1. The changes are described as follows;

Numerous research gaps exist in the classification of thoracic diseases using chest X-rays, despite advances in deep learning [8]. Because patient demographics and imag-ing conditions differ, models frequently do not generalize well across datasets [9]. Pre-dictions are biased as a result of data imbalance, especially when rare diseases are un-derrepresented [10]. The majority of datasets only include labels at the image level, which restricts interpretability and localization [11]. Patients frequently appear with numerous illnesses, making multi-label categorization difficult for current models [12]. Deep learning systems also operate as "black boxes," providing little explanation and casting doubt on clinical reliability [13]. They could also pick up erroneous associations from objects that have nothing to do with illness. Practical implementation is hampered by the lack of real-world evaluation and integration into clinical operations [14]. Furthermore, the development of reliable, equitable, and thorough diagnostic tools is further limited by bias across demographic groups and the underutilization of multimodal and longitudinal data [15, 16]. Building clinically dependable and morally decent AI systems for thoracic imaging requires addressing these problems.

To overcome this limitation, this research introduced a deep learning-based thorax disease classification model. The main goal of this study is to create a deep learning framework that is reliable and accurate for classifying thoracic diseases from chest X-ray images. By combining an EnAE with attention processes, the study aims to enhance diagnostic performance by efficiently extracting important features that capture intricate thoracic anomalies [17]. The research uses advanced data augmentation techniques and the ChWO Algorithm to best choose the most important features, addressing the fundamental difficulty of imbalanced datasets and improving the model's capacity to accurately diagnose both common and unusual diseases [18,19]. Furthermore, the framework uses the IMSTrans model in conjunction with a multi-label classification technique to accurately classify instances that exhibit numerous concurrent states, mirroring actual clinical settings. In order to enhance clinical applicability, the research intends to integrate explainability elements that boost model transparency and credibility among medical professionals [20].

2. The literature review should have more critical analysis of prior work. Rather than merely summarizing existing research, the authors should highlight how their study builds upon and differentiates from previous efforts.

**Response:** Thank you for your valuable feedback. We appreciate your suggestion regarding the critical analysis of prior work. In response to your comment, we have revised the literature review section and included a more thorough and critical analysis of existing research. Rather than simply summarizing previous studies, we have now highlighted how our study builds upon these works, identifies gaps, and differentiates itself by addressing areas that have been underexplored or overlooked in previous literature. The changes are highlighted red colour in the revised research manuscript in section 2. The changes are described as follows;

A CX-Ultranet was designed by [30] for classify and diagnose 13 distinct thoracic illnesses from plain radiography pictures. A multiclass cross-entropy loss function was used within a compound scaling structure, and EfficientNet was used as the baseline model. In order to create reduction cells and add more skip connections, channel shuffling was applied at different points along the network. Together, the loss function algorithm and Adam optimizers stabilize the model and enable ongoing learning from fresh data over time.

A transfer learning method for categorizing radiological abnormalities and respiratory conditions from chest X-rays was designed by [31]. There were 752 X-ray photos from the University Clinical Center of Kragujevac and 191,660 public images in the dataset. Infection, pleural thickening, atelectasis, cardiomegaly, tuberculosis, malignancies, non-viral and COVID-19 pneumonia, and healthy cases were among the disorders it covers. This research, in contrast to others, defines up to 18 illness groups and separates healthy from diseased instances. DenseNet121 with CheXNeXt weights and additional layers for fine-tuning are used in the procedure.

CycleGAN-based preprocessing approach for enhanced lung disease classification in ChestX-Ray14 was designed by [32] that starts with identifying images with artifacts and then uses a CycleGAN model to produce sharper images in order to lessen the noise effect of the artifacts. Channel and spatial attention methods are used by the DenseNet-121 model, which is used for classification, to target certain areas of the image. The model was also updated to incorporate additional data from the dataset, specifically clinical features. Table 1 shows the analysis of recent research work.

**Table 1:** Analysis of Recent Research Work

| **Authors** | **Methods** | **Key contribution** | **Limitations** | **Area under ROC (AUC) (%)** |
| --- | --- | --- | --- | --- |
| Hossain, M. I., *et al.* [21] | ThoraX-PriorNet | Employs attention processes and anatomical prior probability maps. | Restrictions on static priors; potential lack of generalizability across datasets | 84.67 |
| Nawaz, M., *et al.* [22] | CXray-EffDet | EfficientDet is used to classify chest diseases. | Object identification is the main focus; disease-specific interpretability is lacking. | 90.80 |
| Upasana, C., *et al.* [23] | Modified Xception Model | Attention-based categorization of pneumothorax | specifically designed for a certain illness (pneumothorax) | 90.00 |
| Xiao, J., *et al.* [24] | Masked Autoencoders | Unsupervised learning for the aspects of thoracic diseases | High processing costs and little transparency | 82.30 |
| Malik, H., & Anees, T [25] | Multi-modal DL with Cough + Imaging | Diagnoses by combining visual and auditory data. | Separate modalities are handled; fusion is not optimized. | 97.00 |
| Han, Y., *et al.* [29] | ChexRadiNet | Uses radiomics properties to classify X-rays. | Extraction of radiomics can take a long time. | 84.30 |
| Kabiraj, A., *et al.* [30] | CX-Ultranet | Multi-label detection of thoracic diseases with a small network | Accuracy may be sacrificed for efficiency; little focus | 93.00 |
| Geroski, T.,*et al.* [31] | transfer learning with DenseNet121 | For chest X-rays, CNN with transfer learning | Potential underutilization of dataset-specific features | 99.00 |
| Chehade, A.H., *et al.* [32] | CycleGAN | increases image quality by augmentation for lung disease classification | increases model intricacy and the possibility of overfitting | 83.87 |

2.1 Problem Statement

Deep learning for thoracic disease classification and localization has advanced, however there are still a number of important research gaps that highlight the importance and applicability of more study in this area. Numerous current models, like the ResNet-based Triplet-Attention model [29] and ThoraX-PriorNet [21], mostly rely on anatomical priors and attention mechanisms. These methods enhance computing complexity and improve accuracy, but they also introduce dependence on the quality of earlier predictions. Similar to this, models like CXray-EffDet [22] and CX-Ultranet [30] use lightweight backbones like EfficientNet to promote computational efficiency, but they still struggle to handle anatomical changes and fluctuating image quality. Although Vision Transformer-based methods [24] provide better multi-label classification, their high computing requirements limit their scalability. Additionally, the majority of research suffers from inadequate generalizability across various clinical contexts, lack of interpretability [25], and dataset imbalance [23]. Despite recent attempts to use CycleGANs to enhance preprocessing and integrate clinical metadata [32], multimodal data integration is still not completely recognized. Furthermore, transfer learning techniques [31] have the potential to benefit from massive data sets, but fine-tuning for domain-specific subtleties remains a difficult task. Thus, the goal is to create a robust system that improves diagnostic accuracy, reduces false positives and negatives, and supports radiologists in making faster, more accurate decisions, ultimately improving patient outcomes. This research is motivated by the difficulties of correctly identifying thoracic disease from chest X-ray scans, which frequently entail complicated anatomical variances and imbalanced datasets. A novel EnAE with an attention mechanism for robust feature extraction, sophisticated data augmentation techniques to reduce class imbalance, and the ChWO algorithm for optimal feature selection are all used in the proposed approach to address these problems. Finally, the IMSTrans model facilitates accurate classification of thoracic diseases, enhancing precision and facilitating improved clinical decision-making.

3. The manuscript mentions a publicly available dataset but lacks specifics (e.g., class distribution, preprocessing steps like handling missing or noisy data). Clarify these details to ensure reproducibility.

**Response:** Thank you for your valuable feedback. We have now included additional details about the dataset in the manuscript. Specifically, we have provided information on the class distribution, as well as the preprocessing steps taken to handle missing and noisy data. These updates ensure better clarity and reproducibility of our work. The changes are highlighted red colour in the revised research manuscript in section 4.1.2. The changes are described as follows;

**4.1.2 Data Distributions and Preprocessing Steps**

**Dataset 1: ChestX-Ray dataset**

- **Source**: Publicly available NIH ChestX-ray14 dataset.
- **Subset Size Used**: 8,274 chest X-ray images.
- **Classes** (14 in total):
  Atelectasis, Cardiomegaly, Effusion, Infiltration, Mass, Nodule, Pneumonia, Pneumothorax, Consolidation, Edema, Emphysema, Fibrosis, Pleural Thickening, Hernia.
- **Nature**: Multi-label, class-imbalanced dataset.
  Infiltration and Effusion are overrepresented, while Hernia and Fibrosis are rare.
- **Label Handling**: The "Finding Labels" column is parsed to assign binary multi-label vectors for each class.
- **Preprocessing:**
  - Images with missing or unreadable files were removed.
  - Images resized to 256×256, followed by random crop (224×224).
  - Pixel normalization with ImageNet statistics.
  - Missing or null labels were excluded.
- **Data Augmentation**:
  - Random horizontal and vertical flips (p=0.5).
  - Random rotations (up to ±30 degrees).
  - Augmentation increases feature variability but does not alter label distribution.

**Dataset 2: Lung Disease Dataset**

- **Source**: dataset containing **4,260 images**.
- **Classes**: Normal, Pneumonia, and Fibrosis.
- **Nature**: Multi-label, class-imbalanced dataset.
- **Preprocessing:**
  - Images resized and normalized using the same procedure as Dataset 1 to ensure consistency.
  - Verified for label completeness and removed any ambiguous or mislabeled samples.

4. Provide more details on hyperparameter tuning (e.g., learning rates, batch sizes) and any cross-validation strategies used.

**Response:** Thank you for your valuable feedback. In response to the reviewer’s comments, we have included further details on the hyperparameter tuning and cross-validation approaches used. The changes are highlighted red colour in the revised research manuscript in section 4.1.4. The changes are described as follows;

**4.1.4 Hyperparameter Tuning and Cross-Validation:**

Enhancing the input feature space through feature selection based on the ChWO algorithm was the optimization strategy's main objective. By making sure that only the most relevant features were included for classification, this technique successfully decreased feature redundancy, increased class separability, and improved overall model performance.

- **Batch Size and Learning Rate:** To balance memory limitations with training stability, we chose widely used empirically validated settings a fixed learning rate of 1e-4 and a batch size of 32.
- **Optimizer:** The Adam optimizer was chosen because of its demonstrated convergence efficiency in deep learning problems, and it has a weight decay of 1e-5.
- **Training Epochs:** Up to 100 training epochs were used to give the models enough time to converge without being overfit.

5. In discussion section, consider including a comparison of findings with prior studies to highlight the novelty and significance of the work.

**Response:** Thank you for your insightful comment. We have revised the discussion section to include a comprehensive comparison of proposed findings with previous studies in the field. This comparison highlights both the similarities and differences with existing research, thereby emphasizing the novelty and significance of the work. We believe this addition strengthens the overall contribution of the study and provides readers with a clearer understanding of how proposed results advance current knowledge in the field. The changes are highlighted red colour in the revised research manuscript in section 4.7. The changes are described as follows;

**Table 6.** Comparative Analysis of proposed and state-of-the-art methods

| **Dataset 1** | | | |
| --- | --- | --- | --- |
| Methods/ Metrics | Accuracy | Precision | Recall |
| CX-Ultranet [30] | 0.844 | 0.857 | 0.835 |
| DenseNet121 [31] | 0.861 | 0.874 | 0.849 |
| CycleGAN [32] | 0.933 | 0.947 | 0.921 |
| **Proposed (90%)** | **0.964** | **0.977** | **0.9845** |
| **Dataset 2** | | | |
| CX-Ultranet | 0.842 | 0.832 | 0.819 |
| DenseNet121 | 0.867 | 0.869 | 0.842 |
| CycleGAN | 0.949 | 0.956 | 0.942 |
| **Proposed (90%)** | **0.986** | **0.985** | **0.985** |

A comparative analysis of proposed and state-of-the-art methods for classifying thoracic diseases across two datasets is shown in Table 6. The suggested approach achieves the highest accuracy, precision, and recall at 90%, outperforming all others. It considerably outperforms CycleGAN with 93.3% accuracy, 94.7% precision, and 92.1% recall on Dataset 1, achieving 96.4% accuracy, 97.7% precision, and 98.45% recall. Likewise, the suggested approach attains 98.6% accuracy, 98.5% precision, and 98.5% recall in Dataset 2. On the other hand, DenseNet121 and CX-Ultranet perform moderately, demonstrating the superior efficacy of the proposed model in classifying thoracic diseases.

6. Some citations (e.g., [21]–[32]) are formatted inconsistently. Ensure all references follow PLOS ONE guidelines.

"ChextX-Ray dataset" → "ChestX-Ray dataset" (Line # 434).

"publically" → "publicly" (Line # 191, 434).

**Response:** We thank the reviewer for their constructive comments. In response, we have carefully reviewed the manuscript and made the necessary corrections. First, we have ensured that all citations follow the PLOS ONE guidelines for consistency. Additionally, we have corrected the term "ChextX-Ray" to "ChestX-Ray" on line #434, as well as the spelling of "publically" to "publicly" on lines #191 and #434. These changes address the issues raised, and we believe they enhance the clarity and accuracy of the manuscript. The changes are highlighted red colour in the revised research manuscript.
